# Computer literacy and E-learning perception in Cameroon: the case of Yaounde Faculty of Medicine and Biomedical Sciences

**DOI:** 10.1186/1472-6920-13-57

**Published:** 2013-04-19

**Authors:** Georges Bediang, Beat Stoll, Antoine Geissbuhler, Axel M Klohn, Astrid Stuckelberger, Samuel Nko’o, Philippe Chastonay

**Affiliations:** 1 Faculty of Medicine and Biomedical Sciences, University of Yaounde I, P.O. Box 1364, Yaoundé, Cameroon; 2 Department of Radiology and Medical Informatics, University of Geneva, Geneva, Switzerland; 3 Institute of Social and Preventive Medicine, Faculty of Medicine, University of Geneva, Geneva, Switzerland; 4 Unit of Development and Research in Medical Education, Faculty of Medicine, University of Geneva, Geneva, Switzerland

**Keywords:** E-learning, Medical education, Developing Countries, Africa

## Abstract

**Background:**

Health science education faces numerous challenges: assimilation of knowledge, management of increasing numbers of learners or changes in educational models and methodologies. With the emergence of e-learning, the use of information and communication technologies (ICT) and Internet to improve teaching and learning in health science training institutions has become a crucial issue for low and middle income countries, including sub-Saharan Africa. In this perspective, the Faculty of Medicine and Biomedical Sciences (FMBS) of Yaoundé has played a pioneering role in Cameroon in making significant efforts to improve students’ and lecturers’ access to computers and to Internet on its campus.

The objective is to investigate how computer literacy and the perception towards e-learning and its potential could contribute to the learning and teaching process within the FMBS academic community.

**Method:**

A cross-sectional survey was carried out among students, residents and lecturers. The data was gathered through a written questionnaire distributed at FMBS campus and analysed with routine statistical software.

**Results:**

307 participants answered the questionnaire: 218 students, 57 residents and 32 lecturers. Results show that most students, residents and lecturers have access to computers and Internet, although students’ access is mainly at home for computers and at cyber cafés for Internet. Most of the participants have a fairly good mastery of ICT. However, some basic rules of good practices concerning the use of ICT in the health domain were still not well known. Google is the most frequently used engine to retrieve health literature for all participants; only 7% of students and 16% of residents have heard about Medical Subject Headings (MeSH).

The potential of e-learning in the improvement of teaching and learning still remains insufficiently exploited. About two thirds of the students are not familiar with the concept of e-leaning. 84% of students and 58% of residents had never had access to e-learning resources. However, most of the participants perceive the potential of e-learning for learning and teaching, and are in favour of its development at the FMBS.

**Conclusion:**

The strong interest revealed by the study participants to adopt and follow-up the development of e-learning, opens new perspectives to a faculty like the FMBS, located in a country with limited resources. However, the success of its development will depend on different factors: the definition of an e-learning strategy, the implementation of concrete measures and the adoption of a more active and participative pedagogy.

## Background

Academic teaching faces great challenges. The integration and the assimilation of ever changing knowledge, the management of an increasing number of learners and the gradual transition from a “traditional” model (i.e. *ex cathedra* lectures) to an “active” and “interactive” model where the learner is the main actor of his learning process [1,2]. In this perspective, the use of ICT, including Internet, represents a promising approach. Defined as “*the use of new multimedia technologies and Internet to improve the quality of learning by facilitating, on the one hand, the access to resources and services, and on the other hand, exchanges and remote collaboration*” [3], e-learning has been reported as facilitating learning and teaching in different domains [4], including health sciences and medicine [5]. This integration in the field of training in medicine and health sciences is currently witnessing a boom [5]. Although e-learning has been adopted in many educational institutions in the health sciences, there still is a lack of evidence of its effectiveness [6], and even more so in the context of low and middle income countries. In sub-Saharan Africa, the use of ICT by the health training institutions has increased over the past decade [7]. Several initiatives are supported by governments, donors or the civil society [8,9]. Yet most of African countries still have huge deficits in ICT infrastructures, connectivity [7], and qualified human ICT resources.

Over the past decade, the FMBS of Yaoundé, Cameroon [10] has increased its computer equipment and improved the access to Internet on its campus with the support of the ministry of higher education and international academic partners. To date, FMBS has about twenty computers dedicated to public use in the library. The Internet connection is provided to the campus by an optical fiber and distributed via cable or wireless to all the FMSB computers. This Internet connection has a theoretical bandwidth of 1024 kilobits per second. Currently, there is no policy or strategy on e-learning in FMBS. Furthermore, there are no formal educational content for students or medical residents relying on information technology (ICT). However, efforts are made to encourage community members to purchase computers for personal use. In addition, since 4 years, a basic training is delivered on the introduction to computing. Designed for undergraduate students, the objective of this course is to provide them basic computer skills.

This survey investigated the computer literacy of the members of the academic community and their perception of e-learning, as well as its potential contribution to learning and teaching at the FMBS. The latter are two key conditions for the implementation of e-learning within this faculty, aiming at improving the quality of teaching and learning.

## Methods

A cross-sectional study based on a questionnaire was conducted with three different groups of subjects: students (medicine, pharmacy and dentistry), residents (postgraduate training) and lecturers of the FMBS. An adapted version of a questionnaire developed by the Unit of Development and Research in Medical Education at the medical faculty of University of Geneva was applied. The questionnaire was formerly validated by the experts who conducted a similar study for the implementation of e-learning at the faculty of medicine of Geneva. With the administrative agreement of FMBS, the questionnaire was sent out to an opportunistic sample [11]. The research team met members of the FMBS academic community who accepted to participate from when contacted in all sectors and locations, namely: FMBS campus, lecture halls, laboratories, hospitals, and library. The team relied on different leaders (e.g., head of promotions, chief or junior residents) in order to sensitize students and residents for their voluntary participation. Teachers were contacted via letters sent to heads of departments and asked to inform their collaborators about the study. The official address list of the teachers was used to identify them. The questionnaires were then collected, either directly after being completed, during an appointment with a member of the research team, handed to the head of promotion or in some cases by e-mail.

For the academic year 2009–2010, the FMBS was constituted by approximately 1000 students, 275 residents and 160 lecturers. The questionnaire was designed with 89 questions for lecturers and 74 for students and residents. Questions were divided into several sections: access to computers and to Internet; mastery of computer and medical information research strategies; knowledge and perception of e-learning and finally, participants’ profile. Various methods of questioning were proposed: multiple choices, ranking or Likert’s scale type questions.

The data was digitalized through the programme EpiData Entry 3.1 and analysed with the statistical software SPSS 17.0 using descriptive statistics. The following statistical tests were used: the non-parametric Kruskal-Wallis [12] test to compare the three independent groups (i.e., students, residents, lecturers) with respect to their perceived ability to use computer; and the Chi-square test to compare them for others variables. A significant level of p < 0.05 means that at least one of the groups is different from others.

This study was carried out according to the ethical principles of the Helsinki Declaration and in line with the local guidelines for driving research projects (confirmed by a letter N°0034/UYI/FMSB/VDPSAA). All participants were fully informed about the objectives of this study and agreed to voluntarily participate.

## Results

### Response rates

Six hundred (600) questionnaires were distributed, i.e., 400 to students, 110 to residents and 90 to lecturers. Out of these questionnaires, 307 (51%) were completed and analysed: 218 from the students, 57 from the residents and 32 from the lecturers. Unfortunately, some items obtained a low rate response without any correlation with a sub-group of respondents.

### Participant profile

Gender balance was almost reached with 56% of participants being men and 44% being women. Among the group of students, 67% were medical students and 63% were at the bachelor level. Eighty-eight percent (88%) of the residents were in their first, second or third year of specialization. The group of lecturers came from nine academic departments and 53% had a long teaching experience (6 to over 15 years).

### Computer and Internet access

Results show that the majority of students, residents and lecturers use computers and Internet: 78% (170/217) of students, 100% (56/56) of residents and 100% (32/32) of lecturers use computers (p < 0.001), while 49% (105/215) of students, 85% (47/55) of residents and 100% (32/32) of lecturers use the Internet (p < 0.001).

Computers are mainly accessed at home: 76% (63/215) of students, 81% (46/57) of residents and 91% (29/32) of lecturers). Home is the first place to access Internet for residents (61% [35/37]) and lecturers (87% [28/32]) however it is mainly carried out at the cyber café for 49% (105/216) of students.

The majority of participants to the study have a laptop: 53% (116/217) of students, 81% (44/54) of residents and 50% (16/32) of lecturers (p < 0.001). However, it is important to underline that 17% (37/217) of students still do not have a personal computer.

### Computer and information literacy

Participants’ perception of their computer skills was evaluated based on a scale ranging from 1(nil) to 6 (very good). Compared to residents and lecturers, the students reported more confidence in their ability to use a computer (Table 1).

**Table 1:** Computer skills of participants

	Ability with a computer*		
	**Students**	**Residents**	**Teachers**
n	214	57	32
Minimum	1	1	1
Maximum	5	4	4
Median	3	2	2
IQR	2-4	2-3	1-3
Mean	2,76	2,37	1,91
S.D	1,02	0,84	0,96

*The Kruskal-Wallis test was used for the comparison of the three groups: *x*^2^ = 22.09, ddl = 2, P < 0,001.

#### Software used

By far, the software the most frequently used (i.e., several times per week to several times per day) is office software such as text processing (44% [90/204] of students, 68% [37/54] of residents, 93% [29/31] of lecturers, p < 0.001). In comparison, other software such as statistical software are not used frequently (1% [2/192] of the students, 4% [2/52] of the residents, 14% [4/28] of the lecturers, p < 0.001).

#### Internet services

The two most used Internet services in each group are i) the search for medical information used by 41% [84/203] of the students, 57% [30/53] of the residents, 39% [11/28] of the lecturers, and ii) the e-mail service used by 28% [56/203] of the students, 26% [14/53] of the residents, 43% [12/28] of the lecturers.

The FMBS does not offer institutional e-mail addresses. Consequently, only 39% (81/207) of students, 66% (37/56) of residents and 100% (29/29) of lecturers have a private electronic mail box (p < 0.001). For residents (71% [40/56]) and lecturers (48% [14/29]), e-mails are primarily used to communicate with friends.

#### Health information retrieval

Medical information the most searched for on Internet is scientific articles, those searches are conducted by 87% [184/212] of students; 98% [56/57] of residents; 100% [31/31] of lecturers). This result could be linked to the fact that 76% (23/30) of the lecturers frequently recommend to their students (several times per week) to search for further medical information online in order to complete their teaching.

The most frequently used search engine is Google. The Medical Subject Headings (MeSH) vocabulary remains mostly unknown to learners: only 7% (16/215) of students and 16% (9/56) of residents have heard about it, while it is fairly well known among the lecturers (57% [17/30]), p < 0.001).

#### Evidence-based and reliability of health information

The concept of evidence-based medicine is still not well known to students (27% [56/209]) compared to residents (95% [54/57]) and to lecturers (93% [28/30], p < 0.001). Furthermore, participants have little knowledge about accreditation processes of medical web sites, as well as about organizations in charge of it.

### Knowledge and perception of E-learning

#### Current use of ICT for learning or teaching

Although the traditional tools for teaching such as the blackboard, the overhead projector or transparencies are still quite frequently utilized, most participants prefer the use of computer software and constitute 92% [190/207] of the students, 100% [57/57] of the residents, 97% [30/31] of the lecturers, p = 0.056. Among lecturers, 53% (16/30) have never used Internet in the classroom. However, a vast majority (94% [30/32]) are willing to use Internet by making some elements of their teaching available on the Internet.

#### Understanding of E-learning

A majority, about two thirds of the students (131/201) are not familiar with e-leaning, which reveals a significant difference compared to the group of residents and lecturers. The majority of students (84% [158/189]) never accessed e-learning resources, and a little more than half of residents (58% [32/55]).

Three definitions of e-learning were proposed, which were ranked by each group based on their respective relevance.

About two thirds (64% [68/107] of students, 67% [24/36] of residents and 70% [16/23] of lecturers) considered the following definition as the most relevant: “e-learning is the use of ICT and Internet to improve teaching and learning, and to foster exchange and collaboration at a distance”. However, about a third of all groups (29% [31/107] of students, 28% [10/36] of residents and 30% [7/23] of lecturers) considered as most relevant that “e-learning is useful for providing a teaching at a distance without any contact between the lecturer and the student”.

#### Perceived value of E-learning

When asked about the value of e-learning, a majority of students, residents and lecturers consider that e-learning is favouring learning by students in several ways as displayed in Table 2.

**Table 2:** Perceived value of e-learning for the students’ learning

**Variables**	**Students**		**Residents**		**Lecturers**		P*
	**n**	**%**	**n**	**%**	**n**	**%**	
Improves access to educational resources	96/102	94.1	34/39	87.2	21/21	100	0.142
Improves teacher/learner interactions	62/82	75.6	21/32	65.6	13/18	72.2	0.560
Improves collaboration with teacher/learner	62/80	77.5	23/33	69.7	14/17	82.4	0.550
Improves quality of feedback	67/89	75.3	21/30	70.0	14/18	77.8	0.799
Improves methods of personal working	97/102	95.1	32/35	91.4	17/22	77.3	0.22
Develops autonomy in learning	89/97	91.8	33/35	94.3	20/23	87.0	0.614
Develops a learning more suited to our setting	58/95	61.1	22/36	61.1	9/14	64.3	0.973
Develops critical thinking	71/88	80.7	23/33	69.7	13/16	81.3	0.407
Improves creativity	77/93	82.8	24/34	70.6	18/20	90.0	0.162
Develops transversal skills (autonomy, self-assessment, etc.)	81/94	86.2	26/33	78.8	15/19	78.9	0.520

* The chi-square test was used for the comparison of the three groups.

When asked about the value of e-learning tools to facilitate or improve teaching, a majority of lecturers consider it very helpful (Table 3).

**Table 3:** Perceived value of e-learning for teaching (lecturers)

**Variables**	**Frequence**	**Percentage**
Improves preparation and driving of teaching	18/20	90.0
Improves the quality of bibliographic references	18/20	90.0
Enriches the teaching content	20/21	95.2
Improves research and relationship with news	18/19	94.7
Makes educational material available	19/20	95.0
Enables to Archive documents	16/18	88.9
Helps to prepare teaching	16/20	80.0
Improves clarity of the educational material	17/19	89.5
Improves quality of communication	17/19	89.5
Improves follow-up of students activities	16/18	88.9

There is a large consensus among the three groups about the usefulness of specific e-learning resources/tools for learning or teaching at the faculty (Table 4). More specifically, digital learning environments (Learning Management System; LMS) were considered as useful by 94% (166/177) of students, 91% (42/46) of residents and 96% (22/23) of lecturers (p = 0.754). Furthermore, 76% [81/107] of students and 84% [16/19] of residents consider “posting online the documents used for teaching” at the faculty was of prime importance while the lecturers (82% [18/22]) favour practical information (e.g. description, objectives and planning of the teaching, agenda of the main upcoming events).

**Table 4:** Perceived usefulness of specific e-learning resources for learning or teaching

	**Students**		**Residents**		**Lecturers**		P*
	**n**	**%**	**n**	**%**	**n**	**%**	
Printing Device	190/193	98.4	54/54	100	30/30	100	0.517
Online content (text)	179/187	95.7	45/48	93.8	26/28	92.9	0.727
Podcasting	147/165	89.1	37/46	80.4	20/23	87.0	0.299
Interactive educational software	152/174	87.4	31/42	73.8	16/20	80.0	0.082
Tools for self-evaluation	167/178	93.8	45/49	91.8	21/24	87.5	0.507
Computerized simulator of clinical cases	169/177	95.5	46/51	90.2	21/23	91.3	0.317
Communication tools	146/164	89.0	37/44	84.1	15/20	75.0	0.180
Collaborative tools	149/169	88.3	38/42	90.5	20/23	87.0	0.890

* The chi-square test was used for the comparison of the three groups.

## Discussion

This is the first study focusing on e-learning in Cameroon at the university level. The objective was to investigate the integration of ICT and e-learning at the FMBS, in order to facilitate their integration into teaching and learning.

The answer rate to the questionnaire was of 51% of persons contacted. For each of the three groups, it represents a proportion of 20% or more of the total corresponding enrolment group at FMBS: 22% for students, 21% for the residents and 20% for the lecturers. These results provide a global view on ICT utilisation and on the perception of e-learning at the FMBS.

The data collected are based on self-appreciation. While subjective appraisal has been valued as an indicator in many surveys, it also raises the question of potential bias on the objective utilisation and practices studied. Thus it can also be considered reflecting either overestimation or underestimation, which can differ from one group to another. For example, students belong to the Internet generation more than lecturers do [13,14]. They were born when computers and Internet became available throughout the world whereas the lecturers are from a generation who witnessed the emergence of computers and Internet teaching.

Overall, a fairly good mastery and access to ICT, including Internet, was observed in the FMBS community – in all three groups of students, residents and lecturers. Considering these results, we can underline a huge opportunity for a faculty of medicine like the FMBS, located in a country with limited resources. However, major challenges must be faced, among which the rapid knowledge growth and the diversification of training offered in parallel to an increasing number of students with scarce resources yet aiming at high quality of training.

This promising perspective is reinforced by the large consensus of participants to adopt e-learning and “voluntariness” [15] to develop e-learning systems at the FMBS. To note that a low response rate was found for this question compared to the question on access and mastery of information technology. This is probably due to the fact that e-learning is a new concept and most do not know what to answer and thus abstained, and this although the definition of e-learning was clearly given at the beginning of the question to precisely avoid non-responses of the participants and help understanding the concept of e-learning. The low rate could also be due to the assumptions of participants that this was so relevant that it did not need rating.

### Perspectives

Considering the enthusiasm generated by e-learning its implementation, taking into account the local context, requires particular attention in order to avoid the “paradoxal” situations [16], in which the implementation of e-learning would not produce the expected results. This is even more relevant in low-and middle-income setting where infrastructural and human resources are often lacking [17], especially in Africa where specific models should be thought and implemented [18]. Successfully achieving implementation goals can only be done with the will and the involvement of all actors at the faculty. Thus, managers of the institution need to establish a clear e-learning strategy [19] and to take all measures for its sound implementation.

Scientific literature reveals that in order to integrate e-learning in a training curriculum, one has to consider certain key challenges related to technology, to individuals, to educational material, as well as to the context [20].

Concerning technology, the development and dissemination of ICT throughout the world favoured at a large scale the informal integration of ICT at the FMBS; even though the majority report that their favourite place to get access to a computer is their home and access to Internet is the cybercafé. This could be explained on one hand, by the fact that the participants consider the FMBS campus as a place of knowledge acquisition; the understanding and assimilating steps of this knowledge takes place in a more intimate and familiar environment. On the other hand, the insufficient quality of services related to the computers’ access, to Internet or to other services like documents printing or photocopying at the campus, may explain some dissatisfaction as reported in some studies [21,22]. The weakness of technology performance as a barrier for the e-learning has been reported from another low income country context [23]. Moreover, the participants of this study report that they possess fairly good skills in basic use of computing tools. Most of them frequently use a computer, Internet, and private e-mail addresses. Office software, especially text processing software, is the most frequently used software, as illustrated in a study from Nigeria [24]. The same study shows that Internet is also identified as a valuable source of medical information research [24]. However, participants of the study have a rather scarce knowledge of processes and resources available for medical information research online. This study shows that Google engine is the most frequently used programme despite the fact that search engines such as Google are demonstrated to be less appropriate than professional databases like MEDLINE to obtain reliable medical information [25], which is shown to be more accurate and reliable even than Google Scholar [26]. The participants were also found to have little knowledge of international initiatives (i.e., HINARI by WHO) and to favour free access to scientific literature, as has been reported in a study targeting five African countries including Cameroon [27].

With respect to individual learning, the focus should be on critical factors to improve the motivation, the computer’s skills, the strengthening of confidence and support of students as well as teachers [20]. The perception of e-learning being useful and user-friendly as well as personal attitudes are part of the acceptation model of e-learning [28]. Those factors predict the satisfaction of the learners [29,30]. In our study, a majority of students were not familiar with the concept of e-learning and what it refers to. However, when provided with a clear definition and concrete examples, most of them perceived e-learning as useful, with high efficiency potential, as has been reported in the literature [31,32], the majority wished it was further developed at the FMBS.

This positive perception of e-learning is an important result for the future educational development for the Faculty of Medicine and Biomedical Sciences. Furthermore, the development of participant’s computers skills is a key issue. Research demonstrates that human factors such as lack of computer skills, anxiety towards computer usage and personal discipline are critical to the success of e-learning [22,33]. This requires capacity building training programs in computer science, which must be specifically designed and built for the curricula [24]. The peer mentoring strategy for training (i.e., tutoring of students by other students) could also be explored [34]. With the support of the Faculty of Medicine of Geneva University, this type of intervention was experienced at FMBS between 2004 and 2007. Regarding the lecturers in this survey, the mastery of these technologies is a critical factor of the e-learning success [35] and capacity building through continuous medical education using peer mentoring technics calls upon a profound institutional change in the management of many aspects of the teaching methods.

From an educational perspective, the result of our study can only reiterate, as underlined in other studies, the strong need to reflect on the type of pedagogy and to develop the required and adequate support to increase the effectiveness of e-learning [20,23]. E-learning has the potential to foster or reinforce new training strategies around the learner, often designated as “a learner-centered pedagogy”: i.e., a pedagogy based on motivation, information, analysis, interaction and production [1]. On the other hand, specific attention should be given to the “importance of curriculum design for learning performance” [36]. For example, the concept of evidence-based medicine supporting the use of factual data to improve the quality of care remains little known, in particular by students. The application of this concept is very much based on literature published in high income countries, with restricted relevance and adequacy in a Sub-Saharan Africa countries with limited resources and health coverage like in Cameroon [37]. This problem can be solved through the promotion of initiatives aiming at improving both access to biomedical information in Africa [38] (e.g., Open Access Africa [39] or the HINARI Program [40]), and quality and visibility of biomedical information coming from Africa [41].

Moreover, the observed discrepancies between the wishes of learners and those of the teachers about the nature of the contents to be posted on line highlights the need to base these reforms on an integrative approach with both the learners’ specific needs (key stakeholders) as well as those of the teachers. For the FMBS faculty, the implementation of these new approaches could be coordinated by the service in charge of academic affairs in collaboration with all faculty departments.

Finally, the context in which the implementation of e-learning takes place is also of key importance and should be taken into account for the success of such an initiative [20,23]. This requires the establishment of adapted organization and regulations to foster its institutional and socio-cultural anchoring [20]. With respect to the implementation of e-learning, it will be important for the e-learning managers within a specific faculty to establish the mechanisms to ensure important dimensions: the availability of qualified and dedicated human resources, the financing and adequate allocation of resources, and finally, the support of stakeholders (students, teachers, administrators) according to their needs [23]. Since 2006, a conference called "eLearning Africa" [9] brings together educators and e-learning experts in Africa. One of the aims of this conference is to provide an opportunity for African universities to develop partnerships in e-learning. Finally, the very recent Summit of the “Francophonie” in Kinshasa pointed out the relevance of e-learning and launched a new online education programs. All those promising development are signalling the potentially rapid development of e-learning strategies and policies to improve and increase the pace and reach out of medical education in Africa.

## Conclusion

This study contributes to give a global idea of computer literacy and e-learning perception at the FMBS. Most participants have a fairly good mastery of ICT, although good practices about their use remain insufficiently known. The ICT integration at the FMBS is still mostly individually based with little coordination. The huge potential offered by e-learning and e-learning methods in improving teaching and learning still remains largely unexploited at the FMBS, although students, residents and lecturers are convinced of its utility and relevance.

The advances in connectivity and mobile computing (i.e. on smartphones and tablets) and the interest for e-learning opens new perspectives to medical faculties in Africa to develop even new models adapted to the African context such as mobile learning. However, its local development starts with the definition of a clear strategy, the implementation of some key measures as well as the adoption of an educational methodology allowing the learners to be more active in their training while taking account of their specific needs.

## Abbreviations

ICT: Information and Communication Technologies; FMBS: Faculty of Medicine and Biomedical Sciences; MeSH: Medical Subject Headings; LMS: Learning Management System.

## Competing interests

The author(s) declare that they have no competing interests.

## Authors’ contributions

GB: was involved in the conception of the study, the collection of data, the analysis and the interpretation of data, as well as the writing, discussion and revising of this research article. GB has translated the final French version of the paper in English. BS: have been involved in the conception of the study, the analysis and the interpretation of data, the redaction, the discussion and the revising of this research article. AG: was involved in the conception of the study, and the interpretation of data, the writing, discussion and revising of this research article. AK: was involved in the conception of the study, the writing, the discussion and the revising of this research article. AS: was involved in the conception of the study, the revising and editing of this research article. SN: was involved in, the collection of data and the revising of this research article. PC: was involved in the conception of the study, the analysis and the interpretation of data, the redaction, the discussion and the revising of this research article. AG, AK, AS, PC corrected the English version and approved it. All authors have read and approved the final manuscript.

## Authors’ information

GB: Medical Doctor, MSc in Medical Informatics, student in Master of Public Health

BS: Medical Doctor, MPH, Institute of Social and Preventive Medicine, Faculty of Medicine, University of Geneva

AG: Medical Doctor, Professor, Head of Department of Medical Informatics and Radiology, University of Geneva

AK: Medical Doctor, Institute of Social and Preventive Medicine, Faculty of Medicine, University of Geneva

AS: PhD, Institute of Social and Preventive Medicine, Faculty of Medicine, University of Geneva

SN: Medical Doctor, Professor, Vice-Dean in charge of Academic Affairs of FMBS

PC: Medical Doctor, Professor, Head of Institute of Social and Preventive Medicine, Faculty of Medicine, University of Geneva

## Pre-publication history

The pre-publication history for this paper can be accessed here:

http://www.biomedcentral.com/1472-6920/13/57/prepub

## References

[B1] Lebrun M Pédagogie et technologie: en marche vers "l'autrement" Pédagogie médicale 2000 1 45 5323616985

[B2] Taylor D Miflin B Problem-based learning: where are we now? Med Teach 2008 30 742 763 10.1080/01421590802217199 18946818

[B3] CEC Communication from the Commission to the Council and the European Parliament, The eLearning Action Plan: Designing tomorrow's education, COM (172) 2001 Brussels http://ec.europa.eu/education/archive/elearning/annex_en.pdf

[B4] Lepori B Succi C eLearning in Higher Education. Prospects for Swiss Universities. 2nd Report of the Educational Management in the Swiss Virtual Campus Mandate (EDUM) 2003 Lugano http://www.bbaktuell.ch/pdf/bba2844.pdf

[B5] Choules AP The use of elearning in medical education: a review of the current situation Postgrad Med J 2007 83 212 216 10.1136/pgmj.2006.054189 17403945 PMC2600032

[B6] Ellaway R E-learning: is the revolution over? Med Teach 2011 33 297 302 10.3109/0142159X.2011.550968 21456987

[B7] Williams CD Pitchforth EL O'Callaghan C Computers, the Internet and medical education in Africa Med Educ 2010 44 485 488 10.1111/j.1365-2923.2009.03602.x 20518986

[B8] Glen F Shafika I Survey of ICT and Education in Africa. A summary report, based on 53 country surveys 2007 Washington DC: InfoDev/Word Bank [http://www.infodev.org/en/Publication.370.html], last accessed on October 2011

[B9] eLearning Africa [http://www.elearning-africa.com], last accessed on October 2011

[B10] Faculté de Médecine et des Sciences Biomédicales [http://www.fmsb.uninet.cm/index.php], last accessed on October 2011

[B11] Statistique Canada Méthodes d'échantillonnage[http://www.statcan.gc.ca/edu/power-pouvoir/ch13/5214895-fra.htm], last accessed on December 201123616990

[B12] Kruskal-Wallis one-way analysis of variance [http://en.wikipedia.org/wiki/Kruskal%E2%80%93Wallis_one-way_analysis_of_variance], last accessed on November 2012

[B13] Shortliffe EH Health care and the next generation Internet Ann Intern Med 1998 129 138 140 10.7326/0003-4819-129-2-199807150-00017 9669975

[B14] Shortliffe EH The next generation Internet and health care: a civics lesson for the informatics community Proc AMIA Symp 1998 8 14 http://www.ncbi.nlm.nih.gov/pubmed/?term=The+next+generation+Internet+and+health+care%3A+a+civics+lesson+for+the++informatics+community 9929176 PMC2232298

[B15] Zvanut B Pucer P Licen S Trobec I Plazar N Vavpotic D The effect of voluntariness on the acceptance of e-learning by nursing students Nurse Educ Today 2011 31 350 355 10.1016/j.nedt.2010.07.004 20724043

[B16] Guri-Rosenblit S Eight paradoxes in the implementation process of E-learning in higher education Higher Education Policy 2005 18 5 29 10.1057/palgrave.hep.8300069

[B17] Frehywot S Vovides Y Talib Z Mikhail N Ross H Wohltjen H E-learning in medical education in resource constrained low- and middle-income countries Hum Resour Health 2013 11 4 11 10.1186/1478-4491-11-4 23379467 PMC3584907

[B18] Omwenga EA Waema TM Wagacha PW A model for introducing and impementing E-learning for delivery of educational content within the african context Afr J Sci Technol 2004 5 34 36

[B19] Flückiger Y E-learning à l'UNIGE: Stratégie de développement 2009 Université de Genève http://elearning.unige.ch/institution/politique/Rectorat-decision-CoEns.pdf

[B20] Andersson A Grönlund A A conceptual framework for E-learning in developing countries: a critical review of research challenges EJISDC 2009 38 1 16

[B21] Webster J Hackley P Teaching effectiveness in technology-mediated distance learning Acad Manage J 1997 40 1282 1309 10.2307/257034

[B22] Piccoli G Ahmad R Ives B Web-based virtual learning environments: a research framework and a preliminary assessment of effectiveness in basic IT skill training MIS Quaterly 2001 25 401 426 10.2307/3250989

[B23] Awidi IT Developing an E-Learning Strategy for Public Universities in Ghana. Introducing pedagogy-supporting technology in higher education in Ghana requires rethinking institutional policies and lining up stakeholder support EDUCAUSE Quaterly 2008 31 http://net.educause.edu/ir/library/pdf/EQM0828.pdf

[B24] Ajuwon GA Computer and internet use by first year clinical and nursing students in a Nigerian teaching hospital BMC Med Inform Decis Mak 2003 3 10 10.1186/1472-6947-3-10 14498997 PMC222977

[B25] Wang L Wang J Wang M Li Y Liang Y Xu D Using Internet search engines to obtain medical information: a comparative study J Med Internet Res 2012 14 e74 10.2196/jmir.1943 22672889 PMC3799567

[B26] Freeman MK Lauderdale SA Kendrach MG Woolley TW Google Scholar versus PubMed in locating primary literature to answer drug-related questions Ann Pharmacother 2009 43 478 484 10.1345/aph.1L223 19261965

[B27] Smith H Bukirwa H Mukasa O Access to electronic health knowledge in five countries in Africa: a descriptive study BMC Health Serv Res 2007 7 72 10.1186/1472-6963-7-72 17509132 PMC1885254

[B28] Davis FD Bagozzi RP Warshaw PR User acceptance of computer technology: a comparison of two theoretical models Manag Sci 1989 35 982 1003 10.1287/mnsc.35.8.982

[B29] Wu JP Tsai RJ Chen CC An integrative model to predict the continuance use of electronic learning systems: hints for teaching Int J e Learn 2006 5 287 302

[B30] Arbaugh JB Managing the on-line classroom: a study of technological and behavioral characteristics of Web-based MBA courses J High Tech Manag Res 2002 13 203 223 10.1016/S1047-8310(02)00049-4

[B31] Mansoor I Computer skills among medical learners: a survey at King Abdul Aziz University, Jeddah J Ayub Med Coll Abbottabad 2002 14 13 15 12476856

[B32] Buzzetto-More NA Student perceptions of various E-learning components Interdisciplinary Journal of E-learning and Learning Objects 2008 4 http://www.ijello.org/Volume4/IJELLOv4p113-135Buzzetto413.pdf

[B33] Gagnon MP Legare F Labrecque M Fremont P Cauchon M Desmartis M Perceived barriers to completing an e-learning program on evidence-based medicine Inform Prim Care 2007 15 83 91 17877870 10.14236/jhi.v15i2.646

[B34] Samuel M Coombes JC Miranda JJ Assessing computer skills in Tanzanian medical students: an elective experience BMC Publ Health 2004 4 37 10.1186/1471-2458-4-37 PMC514556 15306029

[B35] Hoegerl C John S E-learning in medical education J Am Osteopath Assoc 2010 110 193 194 20386028

[B36] Bhuasir W Critical success factors for e-learning in developing countries: a comparative analysis between ICT experts and faculty Comput Educ 2012 58 843 855 10.1016/j.compedu.2011.10.010

[B37] Chinnock P Siegfried N Clarke M Is evidence-based medicine relevant to the developing world? PLoS Med 2005 2 http://www.ncbi.nlm.nih.gov/pubmed/15916456 10.1371/journal.pmed.0020107 PMC1140939 15916456

[B38] Gathoni N Enhancing access to health information in Africa: a librarian's perspective J Health Commun 2012 17 Suppl 2 18 22 22724668 10.1080/10810730.2012.666628

[B39] Open Access Africa [http://www.biomedcentral.com/developingcountries/events/openaccessafrica], last accessed on November 2012

[B40] HINARI [http://www.who.int/hinari/fr/index.html], last accessed on October 2011

[B41] Goehl TJ Flanagin A Enhancing the quality and visibility of African medical and health journals Environ Health Perspect 2008 116 A514 A515 10.1289/ehp.12265 19079691 PMC2599773

